# Childhood Unpredictability and Smartphone Addiction in Chinese Adolescents: Mediating Role of Self-Concept Clarity and Self-Control and Moderating Role of Psychological Resilience

**DOI:** 10.3390/bs16010085

**Published:** 2026-01-07

**Authors:** Qingqing Li, Mingyang Zhang, Hailan Wang, Wenjing Liu, Yanjing Wang, Zhuoran Li, Zhenrong Fu

**Affiliations:** 1 Key Laboratory of Adolescent Cyberpsychology and Behavior, Central China Normal University, Ministry of Education, Wuhan 430079, China; liqing_psy@ccnu.edu.cn (Q.L.); wangyanjing@mails.ccnu.edu.cn (Y.W.); brick@mails.ccnu.edu.cn (Z.L.); 2 Key Laboratory of Human Development and Mental Health of Hubei Province, School of Psychology, Central China Normal University, Wuhan 430079, China; 3 School of Law, Central China Normal University, Wuhan 430079, China; zhangmy_law@mails.ccnu.edu.cn

**Keywords:** childhood unpredictability, self-concept clarity, self-control, smartphone addiction, psychological resilience

## Abstract

As a distal factor influencing adolescents’ psychological development and behavioral adaptation, the question of whether and how childhood unpredictability is associated with smartphone addiction remains unclear. To address this gap, this study examined the mediating roles of self-concept clarity and self-control, as well as the moderating role of psychological resilience, in the relationship between childhood unpredictability and smartphone addiction. Using a random cluster sampling method, 2262 high school students (51.59% girls; *M*_age_ = 17.83, *SD* = 0.77) were recruited to complete relevant questionnaires. Correlation analyses revealed that childhood unpredictability was negatively correlated with self-concept clarity, self-control, and psychological resilience, and positively correlated with smartphone addiction. Mediation model results indicated that childhood unpredictability contributes to higher smartphone addiction both directly and indirectly through the independent mediating roles of self-concept clarity and self-control and a chained mediation pathway from self-concept clarity to self-control. Moreover, the link between childhood unpredictability and self-concept clarity was moderated by psychological resilience. These findings highlight the critical role and underlying mechanisms of childhood unpredictability in increasing adolescents’ risk of smartphone addiction and emphasize that fostering psychological resilience should be a key target for prevention and intervention efforts aimed at mitigating the adverse effects of childhood unpredictability.

## 1. Introduction

Childhood unpredictability refers to the inconsistency of resources and the presence of violent threats in an individual’s early life environment (Ellis et al., 2009). As a distal factor in adolescents’ psychological development and behavioral adaptation, childhood unpredictability has been found to be significantly associated with impulsive and addictive behaviors in this age group. Smartphone addiction is defined as a behavioral addiction characterized by significant impairment in an individual’s psychological and social functioning due to excessive smartphone use and an inability to control this usage (Liu et al., 2017). With the increasing popularity of the Internet and mobile devices, smartphone addiction has emerged as a widespread phenomenon in society, with a global prevalence rate of 28.3% (Xiong et al., 2021). Research indicates that adolescents are particularly susceptible to smartphone addiction (Choliz, 2012), which is closely associated with a wide range of mental health issues, including anxiety (Geng et al., 2021), loneliness (Bian & Leung, 2015), stress-related symptoms (Samaha & Hawi, 2016), and difficulties in social adjustment (Koss et al., 2025). However, there is a scarcity of research directly examining whether and how childhood unpredictability affects smartphone addiction. Therefore, the current study employed a cross-sectional approach to investigate the potential mediating roles of self-concept clarity and self-control, as well as the moderating role of psychological resilience, in the relationship between childhood unpredictability and smartphone addiction among adolescents.

### 1.1. Childhood Unpredictability and Smartphone Addiction

Existing literature indicates that childhood unpredictability may serve as a risk factor for smartphone addiction. According to the life history theory, individuals must make trade-offs between prioritizing immediate needs and investing in long-term development when allocating their resources and energy. When chronically exposed to harsh and unstable environments during childhood, individuals tend to adopt an adaptive fast life history strategy (Ellis et al., 2009; Lu & Chang, 2019). This strategy leads individuals to prioritize immediate rewards at the expense of long-term goals, resulting in increased impulsivity (Koss et al., 2025) and diminished self-control (Q. Li et al., 2023). Specifically, individuals raised in unpredictable childhood environments are more likely to engage in excessive smartphone use to seek instant gratification. Furthermore, as posited by the compensatory internet use theory (Kardefelt-Winther, 2014), the lack of control stemming from unforeseen circumstances is compensated for by heightened smartphone use. Empirical studies have demonstrated that childhood unpredictability positively predicts addictive behaviors (Lai et al., 2022; M. X. Zhang et al., 2024). For instance, M. X. Zhang et al. (2023) found that childhood unpredictability positively predicted smartphone addiction among adolescents and revealed the mediating roles of anxiety and self-control. Lai et al. (2022) employed a longitudinal design to illustrate the risk posed by childhood unpredictability in the development of smartphone addiction during adolescence. These findings suggest that childhood unpredictability may increase the risk of smartphone addiction, although the specific mechanisms remain to be further explored.

### 1.2. The Mediating Role of Self-Concept Clarity

Self-concept clarity refers to the extent to which all aspects of an individual’s self-concept are clearly defined, as well as the internal consistency and relative stability of one’s self-perception (Campbell et al., 1996). According to the Identity Disruption Model, early adverse experiences hinder children’s ability to integrate their cognitive and emotional responses into a cohesive self-narrative framework, resulting in disruptions to normal identity formation and self-perception (Vartanian et al., 2023). Empirical research indicates that unpredictable events during childhood can significantly impede the identity formation process (Branje et al., 2021), ultimately diminishing an individual’s self-concept clarity (Yang et al., 2024). In contrast, a stable, warm, and supportive family environment can enhance adolescents’ self-concept clarity (Xiang et al., 2022; Q. Li et al., 2025). Self-concept clarity is a crucial indicator of adolescents’ social development and behavioral adaptation. Adolescents with higher self-concept clarity tend to have better-quality social relationships and greater psychological well-being (Becht et al., 2017; Van Dijk et al., 2014). Conversely, individuals with low self-concept clarity often struggle with effective social skills and coping strategies when faced with social conflict (Bechtoldt et al., 2010) and are more likely to experience psychological issues and heightened feelings of loneliness (Wong et al., 2019). According to the compensatory internet use theory, individuals with greater psychological vulnerabilities (e.g., depression, loneliness) are more likely to immerse themselves in the online world as a means of escaping reality or alleviating tension (Grieve et al., 2017; Kardefelt-Winther, 2014). Therefore, self-concept clarity may mediate the association between childhood unpredictability and smartphone addiction.

### 1.3. The Mediating Role of Self-Control

Self-control, defined as the ability to resist immediate temptations and impulses in pursuit of long-term overarching goals, plays a crucial role in optimizing adaptive outcomes across various domains (Baumeister et al., 2007; Q. Li et al., 2023). According to life history theory, individuals raised in unpredictable environments are more likely to adopt faster life history strategies, which lead to increased impulsivity and a preference for immediate rewards over long-term benefits (Z. Lin & Wang, 2015). Exposure to childhood unpredictability reduces opportunities to learn consistent behavioral norms and delayed gratification, directly impairing the development of self-control (J. Li et al., 2024). Empirical studies indicate that family unpredictability (Q. Li et al., 2023) and environmental unpredictability during childhood (Wang et al., 2024) are negatively correlated with self-control abilities. Specifically, Q. Li et al. (2023) found that family unpredictability in childhood negatively predicts self-control in late adolescence, mediated by neural activity in the inferior frontal gyrus—a brain region associated with self-regulation—providing direct neurobiological evidence for this connection. Numerous studies have demonstrated that self-control is strongly associated with dependent and addictive behaviors across various domains, including disordered eating, gaming addiction, and alcohol abuse. It is important to note that adolescents are in a developmental phase during which the socio-emotional system tends to mature while the cognitive control system lags behind (Hofmann et al., 2009). This developmental immaturity hinders their ability to resist the enticing stimuli presented by mobile devices (Gillan & Rutledge, 2021). Given that smartphones offer vivid and attractive stimuli and interactions, adolescents with inadequate self-control are more likely to yield to these temptations, leading to excessive use and dependency. Extensive research has shown that individuals with lower self-control are more vulnerable to developing patterns of smartphone addiction (Błachnio et al., 2023; Coyne et al., 2019). These findings suggest that childhood unpredictability is associated with deficits in self-control development, which in turn confers an elevated risk of smartphone addiction.

### 1.4. The Chain Mediating Effect of Self-Concept Clarity and Self-Control

As cognitive and regulatory constructs of the self, self-concept clarity and self-control are closely linked. According to identity development theory (Erikson, 1968), the formation of adolescent identity is a prerequisite for developing self-regulation and responsible behavior. A clear self-concept provides a stable framework for establishing long-term goals, guiding individuals to resist immediate temptations and maintain self-control (Higgins, 1996). Specifically, individuals with greater self-concept clarity possess a more organized and stable self-related component within their cognitive structure, enabling them to process information more effectively when faced with situations requiring self-control (Light, 2018). Prior studies have shown that self-concept clarity significantly and positively predicts self-control (Q. Li et al., 2025; Xiang et al., 2024), directly confirming the antecedent influence of self-concept clarity on self-control development. Conversely, research indicates that when individuals experience self-uncertainty, they may reduce their investment in long-term goals in favor of more immediate and salient objectives (Light et al., 2018). For instance, when confronted with tempting alternative goals, self-uncertain individuals are more easily distracted from their primary objectives than their self-certain counterparts (Jiang et al., 2023). Moreover, individuals with low self-concept clarity are often more susceptible to external interference (Vartanian, 2009; Lee et al., 2010), which can hinder their self-control efforts. In conclusion, unpredictable events during childhood can impede adolescents’ identity formation and diminish their self-concept clarity, further obstructing the development of self-regulation and potentially leading to smartphone addiction.

### 1.5. The Moderating Effect of Psychological Resilience

Exposure to childhood unpredictability does not necessarily undermine the development of self-concept in all children and adolescents. Research has demonstrated the buffering effect of psychological resilience in mitigating the negative outcomes associated with adverse childhood experiences (Ding et al., 2017; McKay et al., 2022). Psychological resilience is defined as a state-like mental resource characterized by purpose, flexibility, and effectiveness in formulating and maintaining goal-oriented pursuits (Gucciardi, 2017). Researchers have identified two fundamental components of psychological resilience: adversity and positive adaptation (Mukherjee & Kumar, 2016; Fletcher & Sarkar, 2013). Psychological resilience empowers individuals to achieve their goals despite facing obstacles, disruptions, stress, or adversity (Crust et al., 2019; Gucciardi, 2017). According to the protective model of resilience, individuals with high psychological resilience are relatively unaffected by increasing stress (Luthar & Cicchetti, 2000). High psychological resilience can buffer the negative impact of childhood unpredictability on self-concept clarity, thereby interrupting the subsequent transmission of risk and providing a more fundamental protective effect (Masten, 2018). Evidence suggests that psychological resilience enables individuals to integrate adverse experiences into a coherent self-schema (Willis & Burnett, 2016), subsequently reducing the risk of impaired self-control and smartphone addiction (Liu et al., 2025). Uncovering the underlying pathways and protective factors through which childhood unpredictability influences adolescent smartphone addiction can provide theoretical frameworks and practical guidance for prevention and intervention strategies.

### 1.6. The Current Study

Existing research has established a link between childhood unpredictability and smartphone addiction; however, the underlying mechanisms and protective factors remain insufficiently explored. Life history theory provides a foundational framework for explaining how early environmental unpredictability shapes adaptive behavioral strategies and subsequent addictive tendencies. To address these gaps, the present study employed a cross-sectional design to construct a moderated chain mediation model, examining the mediating roles of self-concept clarity and self-control, as well as the moderating effect of psychological resilience on the relationship between childhood unpredictability and smartphone addiction. The novelty of this study lies in two key contributions: it is the first to explore the joint mediating effects of self-concept clarity and self-control through a chained mediation model, and it identifies psychological resilience as a moderator in the pathway from childhood unpredictability to self-concept clarity, thereby offering a novel perspective on protective factors among high-risk adolescent populations. The hypotheses of the current study were as follows: childhood unpredictability would positively predict smartphone addiction (Hypothesis 1); self-concept clarity and self-control would independently mediate the relationship between childhood unpredictability and smartphone addiction (Hypothesis 2); self-concept clarity and self-control would serve as chain mediators between childhood unpredictability and smartphone addiction (Hypothesis 3); and psychological resilience would moderate the relationship between childhood unpredictability and self-concept clarity (Hypothesis 4).

## 2. Materials and Methods

### 2.1. Participants

A cluster random sampling method was employed to recruit study participants from high schools located in Henan, Hubei, and Guizhou Provinces—administrative regions corresponding to China’s central and southwestern geographic zones, respectively. First, we compiled a geographically based roster of all eligible high schools and then used SPSS 26.0 to randomly select the target schools. After negotiating with the administrators of the selected institutions, a detailed testing timeline was finalized, and participant cohorts were defined. Due to the intense academic pressure associated with college entrance examinations for 12th-grade students, the final sampling pool consisted of all enrolled 10th- and 11th-grade students within the selected high schools. Trained graduate students in psychology assisted classroom teachers in distributing a total of 2390 paper-based questionnaires. After excluding data on patterned responses and incomplete responses, 2262 students (94.64% response rate; ages 16 to 19 years, *M*_age_ = 17.83, *SD* = 0.77; 48.41% males) were included in the formal analysis. Regarding monthly household income, 73 (3.2%) of families reported an income of “less than ¥1000”, 651 (28.78%) “¥1001–¥3000”, 881 (38.95%) “¥3001–¥5000”, 452 (19.98%) “¥5001–¥10,000”, 187 (8.3%) “¥10,001–¥20,000”, and 18 (0.8%) “¥20,001–¥40,000”. Each student was assigned a unique code (e.g., examination number) to replace personal identifiers, ensuring participant anonymity. Participants were informed of the study’s purpose, procedures, and potential risks. Prior to data collection, written informed consent was obtained from the parents or guardians of all participants through the school parent-teacher association. Following data collection, questionnaires were screened, and targeted psychological counseling and support services were provided by the school’s professional psychology teachers. All data were stored on encrypted computers, with access restricted solely to the research team to ensure participant privacy and security.

### 2.2. Measures

#### 2.2.1. Childhood Unpredictability

Childhood unpredictability was measured by integrating household unpredictability and parenting unpredictability in the present study (Q. Li et al., 2023). Participants were asked to recall their childhood (5–12 years old) and required to answer the following description about their parents (seven items, “Sometimes parents don’t know what they are talking about when they yell at me”) and growing-up environment at home (three items, “Things in my house are always disordered”). Each item was answered on a 5-point Likert scale ranging from 1 (extremely disagree) to 5 (extremely agree). Items were averaged so that higher scores indicated greater childhood unpredictability. Cronbach’s α coefficient of childhood unpredictability was 0.81 in this study.

#### 2.2.2. Self-Control

Self-control was measured using the Brief Self-Control Scale (Tangney et al., 2004). The scale has 13 items and is divided into five dimensions: impulse control (e.g., “I tend to say things I shouldn’t say”), healthy habits (e.g., “I have a hard time changing bad habits”), resisting temptation (e.g., “I resist temptation well”), focusing on work (e.g., “I have trouble concentrating”), and limiting entertainment (e.g., “I do things that bring me pleasure but are harmful to me”). Participants answered on a 5-point Likert scale ranging from 1 (not at all like me) to 5 (very much like me) to indicate their general self-control ability. Higher scores on this scale indicated greater self-control ability. Cronbach’s α coefficient of self-control was 0.77 in the current sample.

#### 2.2.3. Smartphone Addiction

Smartphone addiction was measured with the Smartphone Addiction Scale (Su et al., 2014), which contains 22 items across six dimensions: withdrawal behavior (e.g., “If my phone isn’t within reach for a while, I often worry about missing a call.”), salient behavior (e.g., “My classmates and friends often say I spend too much time on my phone.”), social comfort (e.g., “I’d rather chat on my phone than communicate face-to-face.”), negative impact (e.g., “One direct consequence of spending time on my phone is that my study efficiency has declined.”), app use (e.g., “I have to open the same mobile app more than three times on the same day.”), and app updates (e.g., “I keep an eye on the latest apps and download them to my phone.”). Each item was rated on a 5-point Likert scale ranging from 1 (extremely disagree) to 5 (extremely agree). Higher scores on this scale indicated more severe smartphone addiction. In this study, Cronbach’s α coefficient of this scale was 0.90.

#### 2.2.4. Self-Concept Clarity

The Self-Concept Clarity Scale (SCCS), developed by Campbell et al. (1996) and modified by Niu et al. (2016), was adopted to measure self-concept clarity in the present study. The scale consists of 12 items (e.g., “In general, I have a clear sense of who I am and what I am”; reverse-scored item: “I often feel confused about my values and beliefs”), using a 7-point Likert-type scale ranging from 1 (strongly disagree) to 7 (strongly agree). Higher scores indicated greater self-concept clarity. The validity and reliability of the Chinese version of the SCCS have been confirmed in Chinese adolescents and early adults (Q. Li et al., 2025; Xiang et al., 2024). In the current study, the Cronbach’s α coefficient of the scale was 0.88.

#### 2.2.5. Psychological Resilience

Psychological resilience was assessed with a brief and well-validated Psychological Resilience subscale from the Psychological Capital Questionnaire compiled by K. Zhang et al. (2008) in the Chinese adolescents. The scale consists of 7 items (e.g., “I can recover quickly when I encounter setbacks”), using a 7-point Likert-type scale ranging from 1 (strongly disagree) to 7 (strongly agree). Higher scores indicated greater psychological resilience. In this study, the Cronbach’s α coefficient of the scale was 0.75.

### 2.3. Statistical Analysis

Statistical analyses were conducted using SPSS 27.0. First, the Harman single-factor test was performed to assess common method bias (H. Zhou & Long, 2004), with the variance extracted by the first factor being less than 40%, indicating the absence of common method bias. Subsequently, descriptive statistics and correlation analysis were conducted. Then, to test Hypotheses 1, 2, and 3, the hypothesized mediating effect of self-concept clarity and self-control were examined using the PROCESS macro for SPSS (Model 6). Finally, to test Hypothesis 4, the PROCESS macro (Model 83) was used to examine the moderating role of psychological resilience in the chain mediation model (Hayes, 2017). Simple slopes analysis was performed to probe conditional effects at high and low levels of psychological resilience. Both models controlled for sex, age, and household income. A bootstrap test with 5000 repeat samplings was conducted to evaluate statistical robustness, with a 95% confidence interval (CI) not encompassing 0 suggesting a statistically significant effect.

## 3. Results

### 3.1. Descriptive Statistics and Correlation Analysis

As shown in Table 1, the means, standard deviations, and Pearson’s correlation coefficients for childhood unpredictability, self-concept clarity, self-control, psychological resilience, and smartphone addiction were presented. Correlation results showed that childhood unpredictability was negatively correlated with self-concept clarity, self-control and psychological resilience, and positively associated with smartphone addiction (all *ps* < 0.01). Additionally, smartphone addiction was negatively correlated with self-concept clarity, self-control, and psychological resilience (*ps* < 0.01). These interrelations among the key variables provided a foundation for the subsequent mediation and moderation analyses.

### 3.2. Testing the Chain Mediating Effects of Self-Concept Clarity and Self-Control

**Figure 1:**
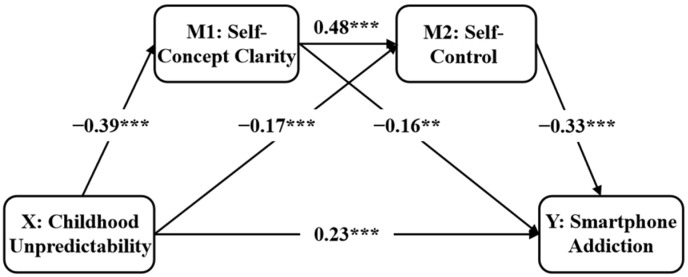
The chain-mediating effect of self-concept clarity and self-control. ** *p* < 0.01; *** *p* < 0.001.

As shown in Figure 1 and Table 2, after controlling for demographic variables (sex, age, and income), the direct predictive effect of childhood unpredictability on smartphone addiction was significant (*β* = 0.41, *p* < 0.001), supporting Hypothesis 1. When the mediating variables of self-concept clarity and self-control were included, this direct effect remained significant (*β* = 0.23, *p* < 0.001). More specifically, childhood unpredictability significantly and negatively predicted self-concept clarity (*β* = −0.40, *p* < 0.001) and self-control (*β* = −0.17, *p* < 0.001). Furthermore, self-concept clarity (*β* = −0.15, *p* < 0.001) and self-control (*β* = −0.33, *p* < 0.001) negatively predicted smartphone addiction, supporting the independent mediating roles of self-concept clarity and self-control (Hypothesis 2). As shown in Table 3, the chain mediating effect was significant (effect value = 0.06, 95%CI [0.05, 0.08]), supporting Hypothesis 3. Based on the model results, the predictive effect of childhood unpredictability on smartphone addiction without mediators was 0.41 (i.e., the total effect value in Table 3), and then this value decreased to 0.23 after incorporating the mediating variables of self-concept clarity and self-control (i.e., the direct effect value in Figure 1 and Table 2). These results indicated that the predictive effect of childhood unpredictability remained relatively stable, while the mediating variable exhibits considerable explanatory power.

### 3.3. Moderated Chain Mediation Test

**Figure 2:**
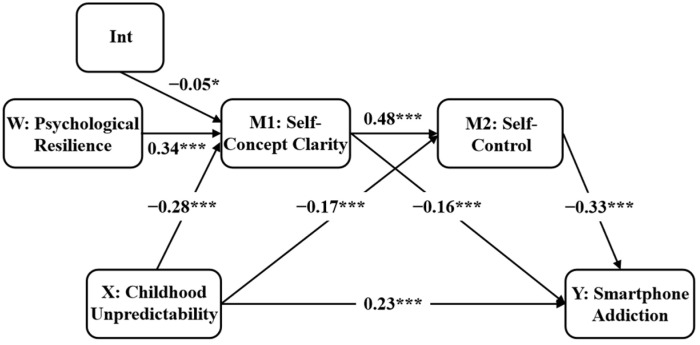
The moderated mediation model. Int = childhood unpredictability * psychological resilience. * *p* < 0.05, *** *p* < 0.001.

After standardizing the model variables, a moderated mediation model was tested to examine the moderating role of psychological resilience in the relationship between childhood unpredictability and self-concept clarity. As shown in Figure 2, results indicated that childhood unpredictability significantly and negatively predicted self-concept clarity (*β* = −0.28, *p* < 0.001), while psychological resilience demonstrated a significantly positive predictive effect on self-concept clarity (*β* = 0.34, *p* < 0.001). Moreover, the interaction between childhood unpredictability and psychological resilience negatively predicted self-concept clarity (*β* = −0.05, *p* < 0.05), supporting Hypothesis 4. This finding indicated that psychological resilience could moderate the link of childhood unpredictability with self-concept clarity.

**Figure 3:**
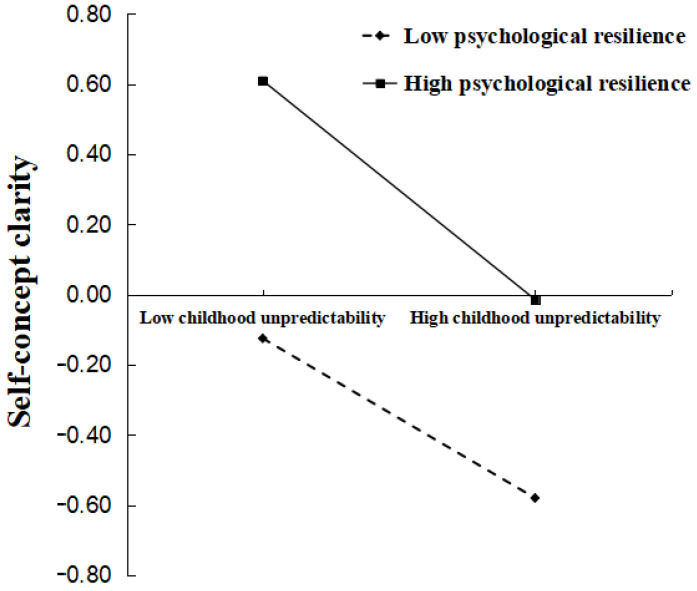
The moderating effect of psychological resilience.

To further elucidate the moderating effect, a simple slope analysis was conducted by evaluating the conditional effects at high (*M* + 1*SD*) and low (*M* − 1*SD*) levels of psychological resilience. As shown in Figure 3, simple slope analysis revealed that in the high psychological resilience group, the negative predictive effect of childhood unpredictability on self-concept clarity was significant (simple slope = −0.34, SE = 0.02, *p* < 0.001). In the low psychological resilience group, this effect was also significant (simple slope = −0.23, SE = 0.02, *p* < 0.001). Moreover, the overall level of self-concept clarity was higher in the high psychological resilience group than in the low resilience group, regardless of childhood unpredictability levels. However, the slope results indicated that, compared to the low resilience group, the high resilience group exhibited a steeper slope, meaning that increased childhood unpredictability led to a more significant decline in self-concept clarity. This finding supports the protective and vulnerability model of resilience, which posits an interaction between stress and personal traits in predicting adaptive outcomes (Luthar & Cicchetti, 2000). Specifically, individuals with high psychological resilience exhibited higher levels of self-concept clarity when facing childhood unpredictability compared to those with low resilience, demonstrating a protective function. Meanwhile, individuals with high psychological resilience were more susceptible to the adverse effects of childhood unpredictability than their low-resilience counterparts, as evidenced by greater declines in self-concept clarity, supporting the vulnerability function.

## 4. Discussion

The present study utilized a cross-sectional approach to construct a moderated chain mediation model that investigated the mediating roles of self-concept clarity and self-control, as well as the moderating effect of psychological resilience in the link between childhood unpredictability and smartphone addiction. The findings indicated that childhood unpredictability predicted higher levels of smartphone addiction both directly and indirectly, through the independent mediating roles of self-concept clarity and self-control, as well as a chained mediation link from self-concept clarity to self-control. Furthermore, the relationship between childhood unpredictability and self-concept clarity was moderated by psychological resilience. These findings underscore the importance and key explanatory mechanisms of childhood unpredictability in increasing the risk of smartphone addiction during adolescence and emphasize that fostering psychological resilience should be a crucial target for prevention and intervention strategies aimed at mitigating the adverse effects of childhood unpredictability.

The positive effect of childhood unpredictability on smartphone addiction provided empirical support for Hypothesis 1. This finding is consistent with previous studies showing that adolescents raised in unpredictable environments are more susceptible to smartphone addiction (Lai et al., 2022; Wang et al., 2024; M. X. Zhang et al., 2024). This finding also aligns with life history theory, suggesting that exposure to a highly unpredictable upbringing leads individuals to adopt fast life history strategies, characterized by cognitive and behavioral tendencies that prioritize immediate gratification and short-term rewards, thereby increasing risk of smartphone addiction. Prior research has shown that individuals who adopt fast life history strategies often exhibit impulsivity, risk-taking behaviors, and addictive tendencies (M. X. Zhang et al., 2024). As an intelligent device integrating multiple functions—including learning, gaming, leisure, and socializing—the smartphone offers adolescents rich, novel, and engaging experiences that cater to their diverse psychological needs. For example, social applications on smartphones facilitate the rapid formation of new social connections, fulfilling adolescents’ desires for short-term social interactions and satisfying their relational needs (H. Lin et al., 2014). Consequently, adolescents who have experienced childhood unpredictability are more likely to become immersed in the immediate feedback associated with excessive smartphone use, which may lead to the development of smartphone addiction.

The mediating effects of self-concept clarity and self-control in the relationship between childhood unpredictability and smartphone addiction supported Hypothesis 2. The finding on self-concept clarity indicates that exposure to childhood unpredictability disrupts the continuity and consistency of adolescents’ self-concepts, increasing the likelihood of smartphone addiction. This aligns with the identity disruption model, which posits that adverse childhood experiences can impede the development of self-identity and reduce motivation for self-exploration. Prior empirical studies have demonstrated that childhood unpredictability—characterized by unstable family dynamics and caregiver instability—contributes to a fragmented self-concept, ultimately leading to addictive behaviors (Yang et al., 2024). The mediating role of self-control indicated that childhood unpredictability obstructed the establishment of consistent behavioral norms and self-regulatory mechanisms, thereby hindering the development of self-control capabilities. Individuals with low self-control become more vulnerable to the immediate gratification provided by smartphones, which can lead to addictive behaviors (M. X. Zhang et al., 2024). These findings suggest that self-concept clarity and self-control are critical determinants in the emergence of smartphone addiction among adolescents raised in unpredictable childhood environments.

The chain mediation effect of self-concept clarity and self-control supports Hypothesis 3, indicating that exposure to childhood unpredictability disrupts self-concept clarity, which in turn impairs self-control, ultimately leading to smartphone addiction. Research has shown that high self-concept clarity enables individuals to identify and prioritize self-initiated, valued goals, thereby facilitating self-regulation in the pursuit of long-term objectives (Higgins, 1996; Light, 2018). Empirical evidence on goal striving and goal pursuit supports this proposition (Jiang et al., 2023; Wong & Vallacher, 2018). For instance, Jiang et al. (2023) found that when individuals feel confused about themselves, they tend to dismiss distal, highly valued goals and prioritize proximal, immediately gratifying goals. Conversely, individuals with low self-concept clarity are more susceptible to external influences, which hinders their self-regulatory processes. In conclusion, self-concept clarity and self-control may serve as critical explanatory mechanisms underlying the relationship between childhood unpredictability and smartphone addiction. This highlights the clinical potential of interventions targeting self-concept clarity and self-control to promote healthy smartphone use.

The moderating results indicated that psychological resilience served both protective and vulnerability functions in the relationship between childhood unpredictability and self-concept clarity, thereby partially supporting Hypothesis 4. The finding on the protective effect of resilience on self-concept clarity suggests that adolescents with high psychological resilience possess greater cognitive flexibility, coping skills, and social support (Masten, 2018, which allow them to integrate negative experiences stemming from childhood unpredictability into a coherent self-schema (Meggs et al., 2014; Ellis et al., 2009). Therefore, they are less likely to experience confusion about their self-identity and are better able to maintain a stable and consistent self-concept. In contrast, adolescents with lower psychological resilience lack sufficient psychological resources to counteract the disruptive effects of childhood unpredictability on self-identity formation, resulting in lower self-concept clarity (Campbell, 1990; M. X. Zhang & Wu, 2022). The finding on the vulnerability function of resilience on self-concept clarity suggests that when confronted with high childhood unpredictability, the stable self-narrative of highly resilient individuals is completely disrupted, leading to greater discrepancies in self-perception and manifesting as vulnerability. In contrast, the self-perception of less resilient individuals is already fragmented, resulting in a smaller decline in self-concept clarity.

The present findings revealed the mechanisms underlying the relationship between childhood unpredictability and smartphone addiction, providing valuable insights for future research on adolescent addictive behaviors. In terms of family education, parents should enhance the stability and predictability of the parenting environment by maintaining consistent parenting styles, establishing clear household rules, and avoiding frequent changes in living and educational settings to promote adolescents’ development of a clear self-concept and strong self-control (Everri et al., 2015). In families characterized by high environmental unpredictability (e.g., single-parent households or families with frequently absent parents), parents should pay greater attention to their children’s psychological needs, strengthen emotional communication, and help children build a stable self-identity (Soenens et al., 2017). Moreover, given the challenges of restructuring family systems, school-based interventions focused on psychological resilience hold greater practical significance and applicability. Studies have shown that resilience-focused interventions can improve the psychological and behavioral adjustment of adolescents exposed to childhood adversity (Carlon et al., 2025; Sanders et al., 2020). Future research could improve self-concept clarity and reduce addiction problems among children raised in unpredictable environments by designing interventions that strengthen psychological resilience.

The present study offers novel insights into the psychological mechanisms linking childhood unpredictability to smartphone addiction through a moderated chain mediation model. However, several limitations must be acknowledged when interpreting the findings. First, the sample was limited to high school students from three Chinese provinces. Although no studies have explicitly examined differences among these provinces, some surveys indicate distinct cultural and economic disparities. For example, compared to Guizhou Province, Henan and Hebei—located in central China and deeply influenced by Confucian traditions—exhibit more hierarchical family structures, which may amplify the impact of childhood unpredictability (Baumrind, 1971). Furthermore, compared to Hubei Province, Guizhou and Henan are major labor-exporting provinces, potentially resulting in more instances of parental separation across these regions. This situation increases the risk of smartphone addiction among left-behind children and adolescents (Wu et al., 2024). Future research should consider potential differences in economic, cultural, educational, and other contextual factors across the regions where participants reside. Second, reliance on self-report measures from a single informant introduces the risk of common method variance and potential biases. Although this study employed the Harman single-factor test to assess common method bias, this approach has inherent limitations. Specifically, it cannot fully account for method variance that is construct-specific, nor can it distinguish between true covariance and method-induced covariance in complex models. Future research would benefit from incorporating complementary data collection methods, such as device usage data, third-party reports (e.g., from parents or teachers), and experience sampling methods, to provide more objective and comprehensive insights into smartphone addiction and its predictors. Finally, the cross-sectional design of this study cannot establish causal relationships among self-concept clarity, self-control, and smartphone addiction. Future research should employ longitudinal designs to track the developmental trajectories of childhood unpredictability, self-concept clarity, self-control, and smartphone addiction over time.

## 5. Conclusions

This study elucidates the psychological mechanisms underlying the relationship between childhood unpredictability and smartphone addiction by examining the mediating roles of self-concept clarity and self-control, as well as the moderating role of psychological resilience. Specifically, adolescents exposed to childhood unpredictability tend to exhibit diminished self-concept clarity, which is associated with reduced self-control and, consequently, an increased risk of smartphone addiction. Psychological resilience mitigates the adverse association of childhood unpredictability with self-concept clarity. Theoretically, these findings enhance our understanding of both the explanatory and protective mechanisms underlying the relationship between childhood unpredictability and smartphone addiction. Practically, they provide a foundation for developing targeted intervention strategies aimed at reducing smartphone addiction by improving the stability of the child-rearing environment through family education and by cultivating psychological resilience in adolescents. Overall, this study underscores the significance of these psychological factors in supporting adolescents’ development of healthy smartphone usage habits.

## Figures and Tables

**Table 1:** Descriptive analysis and bivariate correlations (*N* = 2262).

Variables	*M*	*SD*	1	2	3	4	5
1 Childhood unpredictability	2.49	0.62	-				
2 Self-concept clarity	3.80	1.07	−0.40 **	-			
3 Self-control	2.81	0.57	−0.37 **	0.56 **	-		
4 Psychological resilience	4.11	0.98	−0.33 **	0.45 **	0.50 **	-	
5 Smartphone addiction	2.33	0.60	0.41 **	−0.43 **	−0.51 **	−0.41 **	-

Note: ** *p* < 0.01.

**Table 2:** Regression analysis of variables in the model (*N* = 2262).

Independent Variable	Dependent Variable								
	Self-Concept Clarity			Self-Control			Smartphone Addiction		
	*β*	*t*	95%CI	*β*	*t*	95%CI	*β*	*t*	95%CI
Sex	−0.25	−6.39 ***	[−0.33, −0.17]	−0.08	−2.24 *	[−0.15, −0.01]	−0.03	−0.77	[−0.10, 0.04]
Age	0.01	0.84	[−0.01, 0.03]	0.01	0.90	[−0.01, 0.03]	−0.02	−1.85	[−0.03, 0.00]
Income	0.03	1.54	[−0.01, 0.08]	0.01	0.36	[−0.03, 0.04]	0.01	0.65	[−0.03, 0.05]
Childhood unpredictability	−0.39	−20.44 ***	[−0.43, −0.35]	−0.17	−9.35 ***	[−0.21, −0.14]	0.23	11.84 ***	[0.19, 0.27]
Self-concept clarity				0.48	25.63 ***	[0.45, 0.52]	−0.16	−7.21 ***	[−0.20, −0.11]
Self-control							−0.33	−15.74 ***	[−0.38, −0.29]
R^2^	0.18			0.34			0.34		
F	121.15 ***			232.84 ***			185.32 ***		

Note: * *p* < 0.05; *** *p* < 0.001.

**Table 3:** The mediating effect of self-concept clarity and self-control (*N* = 2262).

Effect	Pathway	Value	SE	95%CI
Direct effect	childhood unpredictability→smartphone addiction	0.23	0.02	[0.19, 0.27]
Mediation effect	childhood unpredictability→self-concept clarity→smartphone addiction	0.06	0.01	[0.04, 0.08]
	childhood unpredictability→self-control→smartphone addiction	0.06	0.01	[0.04, 0.07]
	childhood unpredictability→self-concept clarity→self-control→smartphone addiction	0.06	0.01	[0.05, 0.08]
Total effect		0.41	0.02	[0.37, 0.45]

## Data Availability

Data is available upon reasonable request.
